# The Echo Report Understood: Improving Education in Echocardiography for Junior Doctors

**DOI:** 10.7759/cureus.65630

**Published:** 2024-07-29

**Authors:** Molly M Nichols, Jane Draper, Jessica Webb

**Affiliations:** 1 Cardiology, Guy’s and St Thomas’ NHS Foundation Trust, London, GBR

**Keywords:** postgraduate medical education (pgme), medical education, internal medic (cgm), echocardiography-heart failure-valvular heart disease, undergraduate and graduate medical education, foundation year 1 doctor, clinical diagnostics, echocardiography diagnosis, echocardiography findings, echocardiography education

## Abstract

With echocardiography standing as the most widely used cardiac imaging modality, echocardiography report interpretation is a core responsibility of junior doctors. The literature, however, reveals a deficit in echocardiography education. The implications of this for patient care should not be ignored.

To address this need, a hybrid teaching session was developed for junior (intern and resident grade) doctors, with the aim to increase understanding of echocardiography and increase confidence in report interpretation. Pre- and post-session data were analysed.

Results revealed that the vast majority of respondents received less than an hour of echocardiography teaching at medical school, with over two-thirds receiving less than an hour in the postgraduate setting. A total of 80% of doctors interpreted echocardiography reports weekly, with almost all doctors perceiving this skill as important. Despite this, an overwhelming majority of doctors did not feel confident interpreting reports. The educational intervention achieved significant increases in perceived understanding of echocardiography and confidence with report interpretation. Participants were better able to identify cardiac pathology and understand report terminology.

This intervention has the scope to improve patient safety through better management of cardiac patients and recognition of pathology from echocardiography. This work also identifies a need for more echocardiography education, having uncovered a concerning lack of confidence amongst junior doctors and an appetite for further teaching on this important topic.

## Introduction

As a quick, noninvasive and cost-effective diagnostic and monitoring tool, echocardiography is the most widely used cardiac imaging modality [1]. A familiarity with echocardiography reports is essential for junior doctors’ day-to-day practice, particularly as patient communication of the report result is recognised to be the responsibility of the referring clinician, rather than the sonographer [2]. Despite this, it has been acknowledged that echocardiography education is lacking at medical school [3], with referring doctors often feeling underqualified to interpret the reports [4,5]. A poor understanding may lead to inappropriate referrals, inaccurate report interpretation, and unsafe patient management.

This quality improvement initiative aims to gain an understanding of the quantity and nature of echocardiography education received at medical school, in Foundation Year 1 (FY1: internship) and Internal Medical Training (IMT: residency) while developing the understanding of echocardiography amongst junior doctors through the delivery of high-quality, hybrid echocardiography teaching, with the potential to improve diagnostic stewardship and patient safety.

## Materials and methods

**Figure 1:**
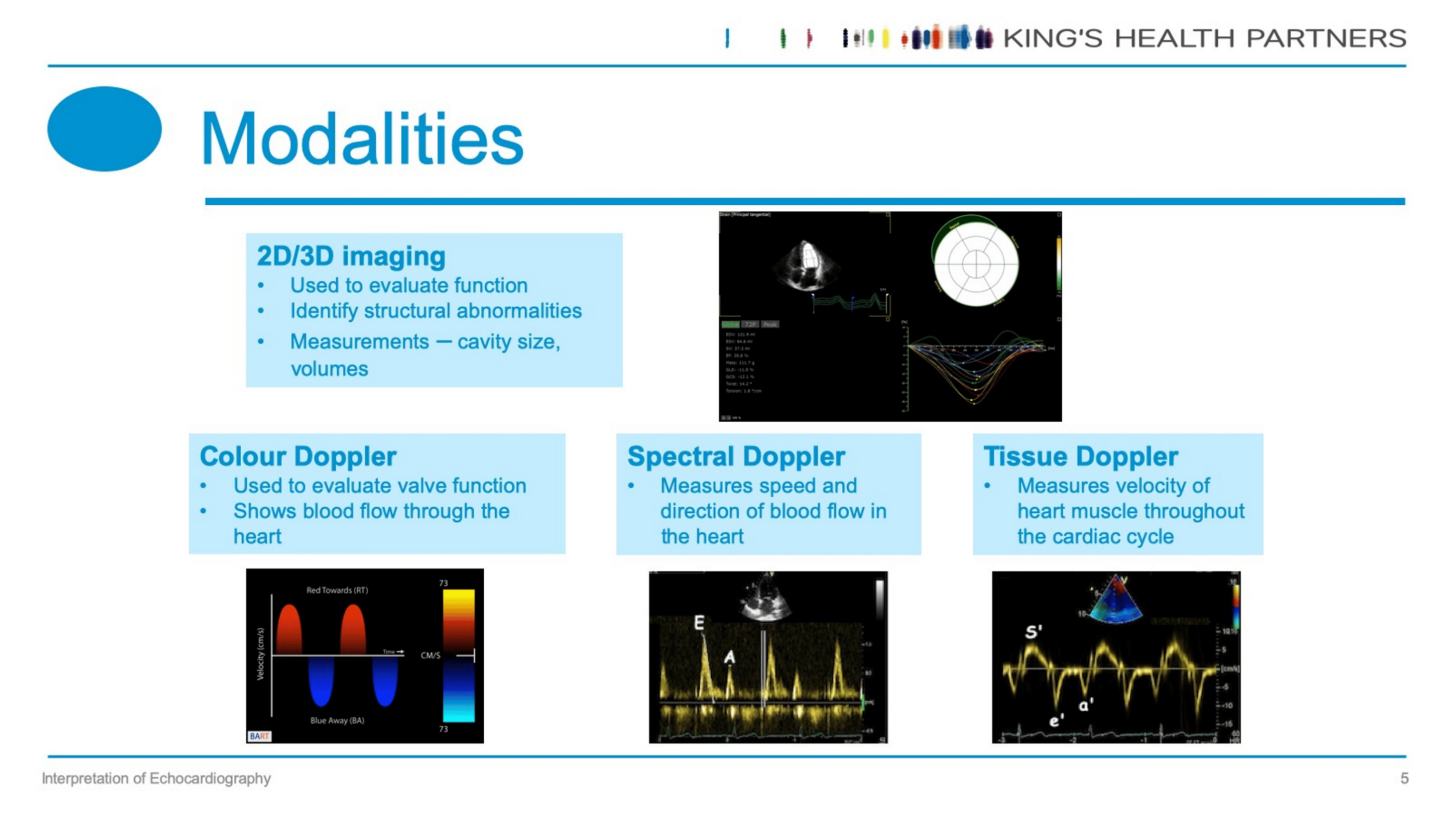
Example presentation slide displaying differing echocardiography modalities

This intervention involves a hybrid teaching session, available to FY1 (internship grade) and IMT (residency grade) cohorts during an hour of protected mandatory teaching at Guy's and St Thomas' Hospitals, an NHS tertiary centre in London. All FY1 and IMT doctors were emailed by the Trust administrative team, inviting them to attend their respective teaching sessions face-to-face or online. The interactive lecture was designed by multidisciplinary members of the cardiology faculty. Content includes echocardiography imaging modalities (Figure 1), report terminology (Table 1), interpretation of echocardiography images and reports, diagnosis and management of common cardiac conditions, and the identification and management of cardiology emergencies. Key-feature questions (KFQs) were used to test the application of knowledge, a tool validated in the assessment of clinical reasoning [6]. In such questions, participants are asked to anonymously select the most appropriate management plan after reviewing clinical vignettes alongside echocardiography videos and reports. 

**Table 1:** Echocardiography report jargon explained in presentation slides

Relevant Structure	Report Terminology	Interpretation
Left ventricle	EF	Ejection fraction: estimates global systolic function of the left ventricle
	RWMA	Regional wall motion abnormality: describes the function of left ventricle segments
	E/E’	Ratio of early diastolic mitral inflow velocity to the early diastolic mitral annulus velocity: estimates left ventricle end-diastolic filling pressure
	LVH	Left ventricular hypertrophy: patterns of hypertrophy may be described. Concentric: consistent with hypertension or infiltrative processes. Asymmetrical or apical: possible cardiomyopathy. Basal septal bulge: focal thickening, normal variant particularly in the elderly
Right ventricle	TAPSE	Tricuspid annular planar systolic excursion: gives information on systolic function of the right ventricle
	PASP (mmHg)	Pulmonary artery systolic pressure: measured from tricuspid regurgitation, evaluates the presence of pulmonary hypertension
Valves	EOA	Effective orifice area: used to grade severity of valvular stenosis
	Peak and mean gradients (mmHg)	Measures the pressure drop caused by stenosis to help grade severity
Right atrium	RAP	Right atrial pressure: estimated by size of right atrium and reactivity of the inferior vena cava

Prior to the intervention, an anonymised baseline assessment was provided for doctor participants to establish their level of confidence and perceived understanding of echocardiography using a Likert scale (see appendix, Figures 6-9). Data were also collected via multiple choice questions on the quantity, quality, and nature of echocardiography education in medical school and the postgraduate setting. A group of final-year King's College London medical students additionally completed this survey. Data were collected from six students, 10 FY and six IMT doctors. A post-session anonymised, online questionnaire assessed the impact of the intervention (see appendix, Figures 10-12). Quantitative data were collected from 14 FY and eight IMT doctors on their perceived understanding and confidence, using a Likert scale. Qualitative feedback was collected in free-text format. All data were collected over a five-month period.

Pre-session and post-session numerical data were statistically analysed using students t-test. No responses were excluded from analysis.

The work was conducted in compliance with the ethical principles of the Declaration of Helsinki [7]. There was no potential harm to participants; anonymity of participants is guaranteed; anonymous data have been collected and stored in accordance with institutional data protection guidelines; and informed consent of participants was obtained for participation and publication. The data collected formed part of the quality assurance and service evaluation processes for medical education at the Trust.

## Results

**Figure 2:**
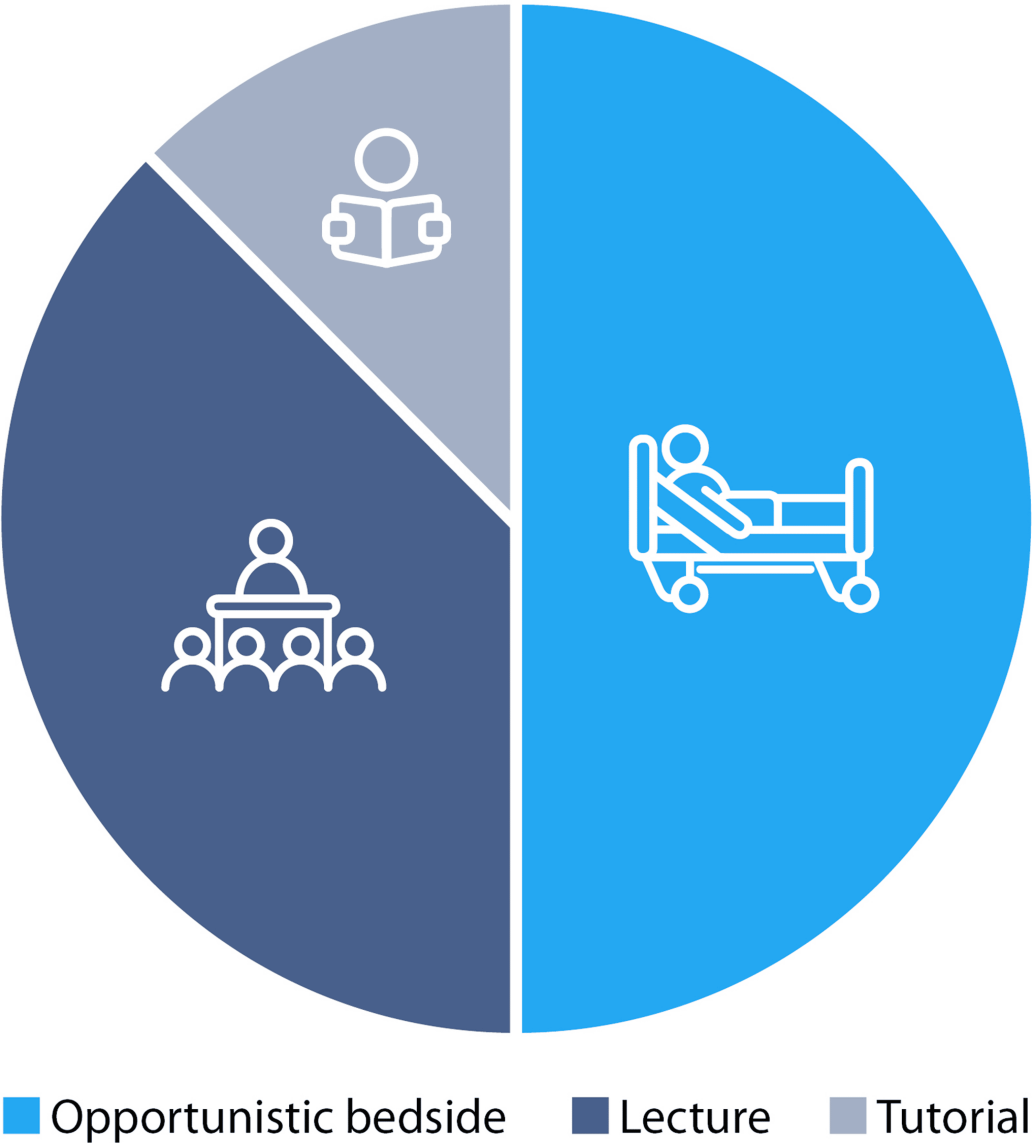
Format of echocardiography teaching received prior to the teaching session (medical student, FY1, and IMT responses combined) FY1: Foundation Year 1; IMT: Internal Medical Training

Analysis of pre-teaching data revealed that 18 (82%) respondents attending 10 different British medical schools received less than one hour of echocardiography teaching during their undergraduate studies. Doctors received a modal average of one hour of teaching following graduation; the FY cohort received significantly less than the IMT cohort (18 vs. 130 minutes, respectively). The format of all teaching received is shown in Figure 2. Within the FY cohort, lecture format was most commonly reported (n = 5, 50%), while most IMT doctors (n = 5, 83%) received opportunistic bedside teaching.

**Figure 3:**
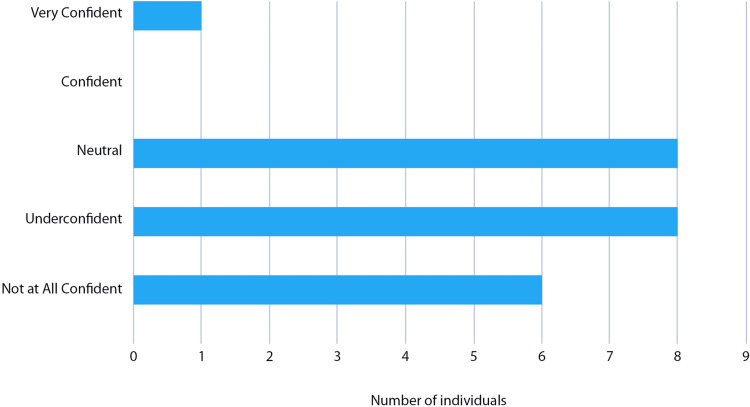
Likert confidence rating with echocardiography report interpretation pre-teaching session (FY and IMT cohorts combined) FY1: Foundation Year 1; IMT: Internal Medical Training

There was no significant difference in the mean frequency of echocardiography report interpretation between FY and IMT groups, with 88% (n = 14) interpreting reports at least once a week. Ninety-four percent of doctors (n = 15) and 83% of students (n = 5) perceive this skill as important. All students interpret reports less than quarterly (n = 6, 100%). All students surveyed (n = 6, 100%) reported that they were ‘not at all confident’ interpreting echocardiography reports and identifying an emergency from a report. On average, doctors rated their baseline confidence between ‘under confident’ and ‘neutral’ (Figure 3). Compared to doctors, medical students were significantly less confident with these skills (p < .001 and p = .005, respectively). This being said, only 6% of junior doctors (n = 1) surveyed felt confident interpreting reports, and 73% (n = 12) reported a poor understanding of report components. When reviewing an echocardiography report, just 20% of doctors (n = 3) felt confident identifying pathology and 13% (n = 2) felt confident identifying a cardiology emergency. Only 19% (n = 3) reported feeling confident knowing the next steps to take after interpreting a report, and none felt confident managing cardiac emergencies identified on a report. 

Of the echo components assessed: ejection fraction (EF), tricuspid annular plane systolic excursion, effective orifice area (EOA), septal bulge, inferior vena cava inspiratory collapse, and the ratio of early diastolic mitral inflow velocity to the early diastolic mitral annulus velocity (E/E’), the best understood was EA, with significantly higher reported understanding than all other components assessed (p < .0001). E/E’ and EOA were the most poorly understood. 

Doctors felt significantly more confident identifying heart failure on echocardiography (p < .0001) than other pathologies assessed (myocardial infarction, new-onset valvular regurgitation, severe aortic stenosis and cardiac tamponade). Identification of cardiac tamponade and new-onset valvular regurgitation had the lowest confidence ratings from FY and IMT cohorts, respectively. On average, doctors felt confident managing heart failure with no significant difference noted between groups (p = .1698).

Within the teaching session, there was no significant difference in performance in the KFQs between FY1 doctors and IMT doctors, with 65% (n = 68) and 55% (n = 42) correct answers, respectively (p = .299).

**Figure 4:**
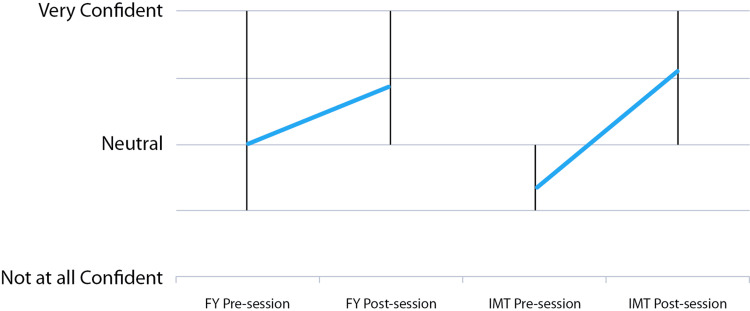
Pre- and post-session self-rating confidence with echocardiography report interpretation in FY1 and IMT doctor cohorts (vertical bars denote the range)

Following the session, there were significant 38% and 77%, respectively, increases in confidence with report interpretation for FY and IMT doctors (p = .0018 and p = .0001, respectively) (Figure 4). 

**Figure 5:**
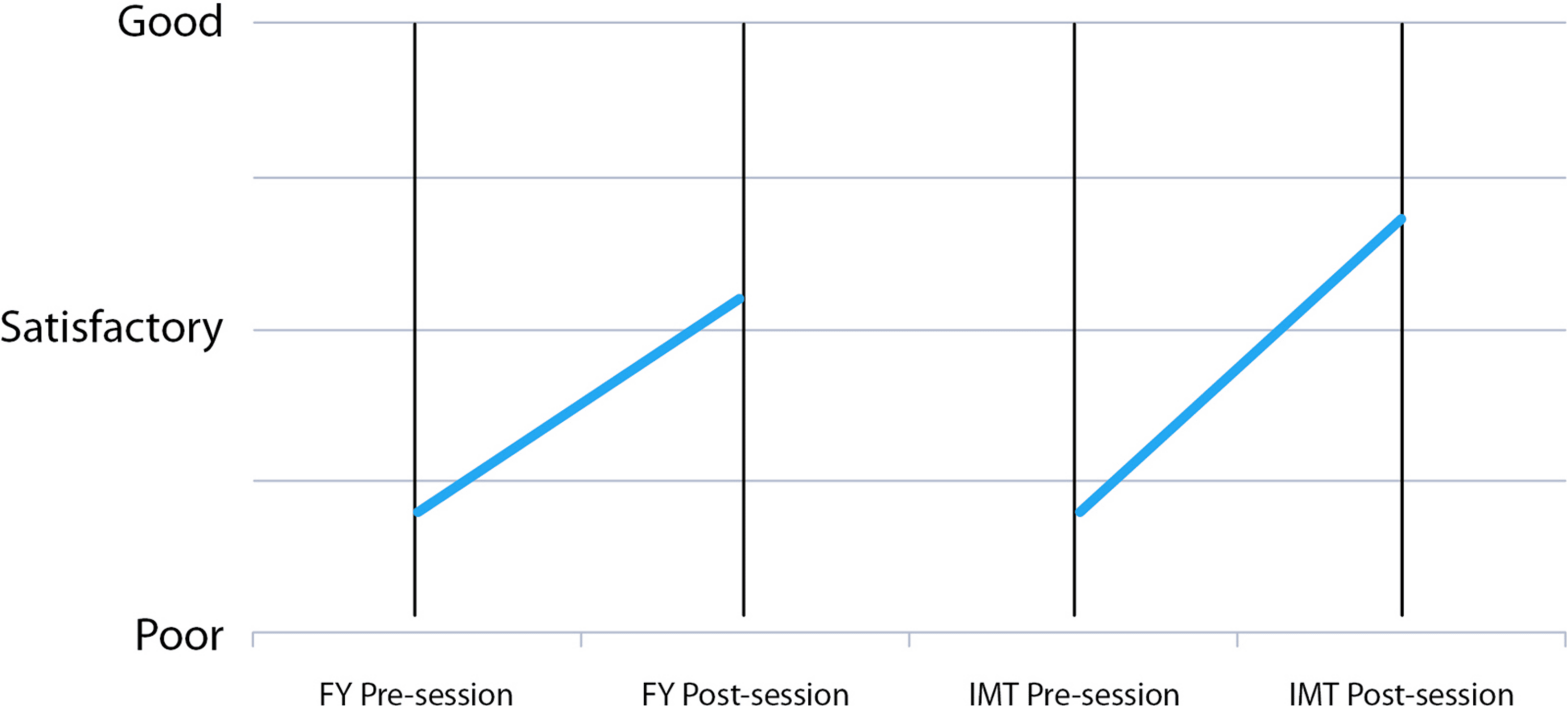
Pre- and post-session self-rating understanding of echocardiography report components in FY1 and IMT doctor cohorts (vertical bars denote the range)

**Figure 6:**
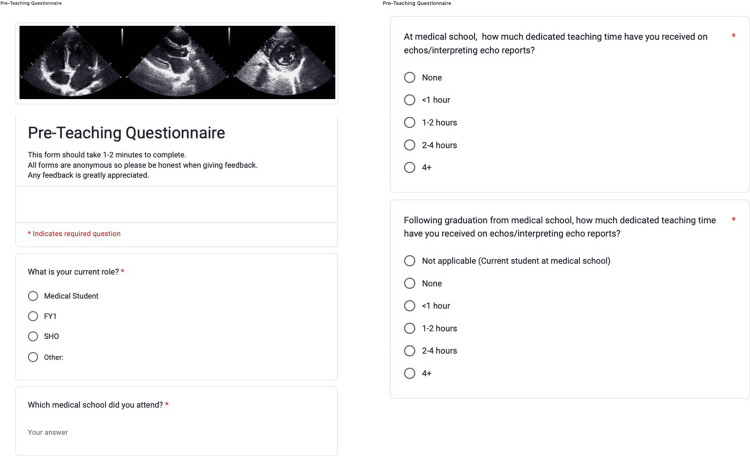
Pre-session survey 1/4

**Figure 7:**
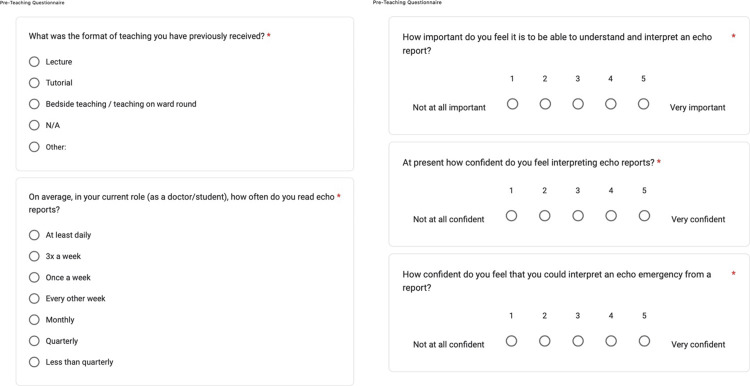
Pre-session survey 2/4

**Figure 8:**
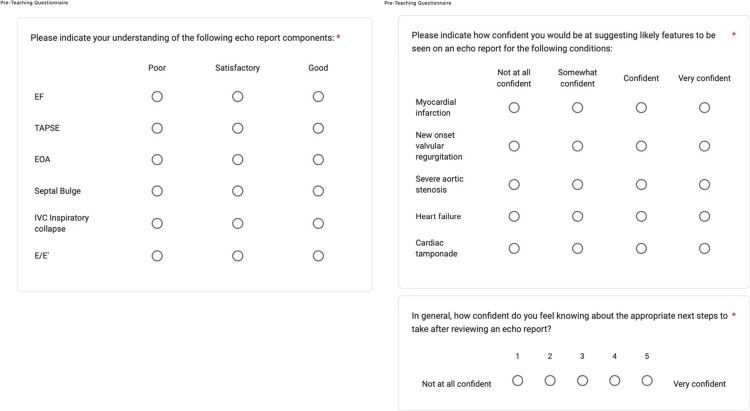
Pre-session survey 3/4

**Figure 9:**
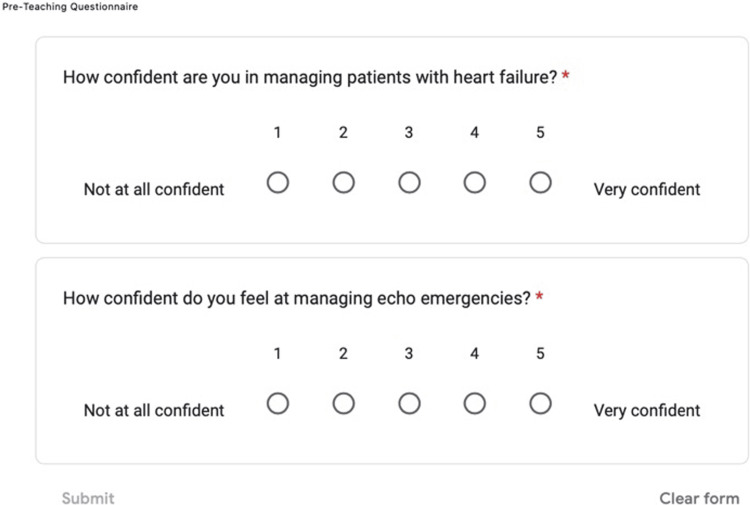
Pre-session survey 4/4

**Figure 10:**
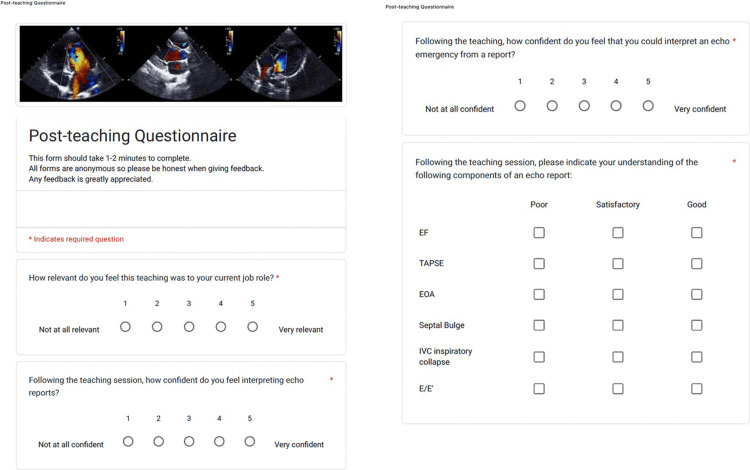
Post-session survey 1/3

**Figure 11:**
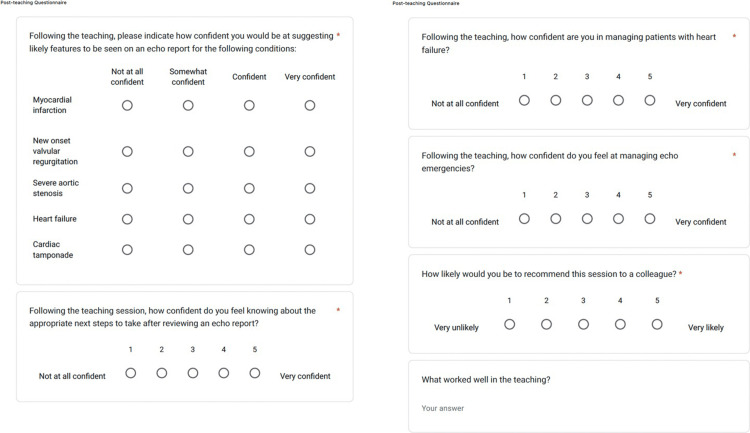
Post-session survey 2/3

**Figure 12:**
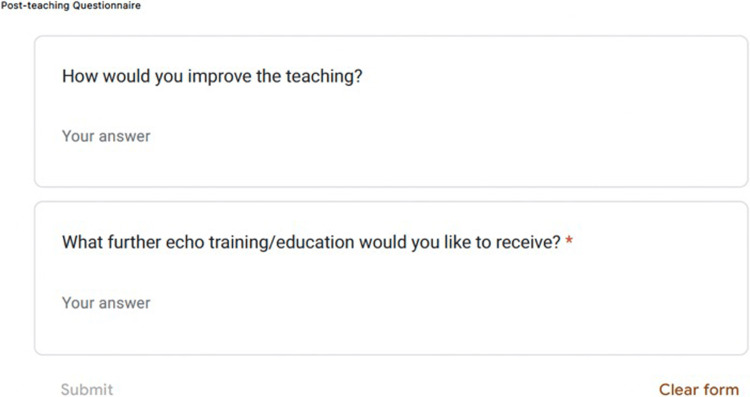
Post-session survey 3/3

Perceived overall understanding of echocardiography report components increased significantly, for FY and IMT doctors (p < .001), with an increase in mean understanding by 50% vs. 78%, respectively (Figure 5). There was a significant increase in perceived understanding of all components in the IMT group; however, there was no significant increase in understanding of EF amongst FY doctors. 

There was an overall significant increase in confidence (p < .001) identifying all pathologies on echocardiography except heart failure. Regarding management of heart failure, there was significant increase in confidence amongst both groups of FY and IMT doctors (p = .003 and p = .04, respectively).

Significant post-session increases in confidence were noted for doctors in knowing the next step to take following echocardiography report interpretation (p < .001), identifying cardiac emergencies from a report (p < .001), and managing such emergencies (p < .001).

Thematic review of qualitative data highlighted a positive response to the case-based discussions, KFQs and interactivity, with 91% of doctors (n = 20) recommending the session to a colleague. Participants requested take-home resources summarising content covered. Most doctors requested further teaching on echocardiography, with lecture-based and practical skills sessions suggested. A number of respondents requested a session covering specialised echocardiography such as trans-oesophageal, bubble, and stress echocardiography. 

## Discussion

Although echocardiography report interpretation is a common task amongst junior doctors, there appears to be limited echocardiography education in the undergraduate and postgraduate settings, reflecting findings in the literature [3]. The relative lack of confidence and perceived understanding of echocardiography reports is likely secondary to this. 

The General Medical Council’s ‘Outcomes for Graduates’ [8] and Medical Schools Council Assessment Alliance ‘Medical Licensing Assessment content map’ [9] fail to specifically mention echocardiography which may result in the topic's omission from local undergraduate curricula. Results of a 2023 systematic review indicated that ultrasound teaching in UK medical schools is not widely reported and highlighted the current lack of a centralised ultrasound curriculum [10]. IMT doctors surveyed received the majority of their echocardiography education as opportunistic bedside teaching, perhaps highlighting a lack of structured education on the topic. FY doctors received most of their limited teaching as lectures; the COVID pandemic may have reduced bedside teaching due to altered patterns of clinical exposure and reduced educational opportunities [11]. Without pre-pandemic data, this would be difficult to establish. 

It is somewhat unsurprising that medical students viewed echocardiography reports less than junior doctors given their limited clinical exposure, however with report interpretation being a common ward task, more undergraduate exposure would better prepare students for the workplace. A 2018 scoping review of ultrasound education amongst medicine undergraduates supported the early introduction of such teaching, concluding that it is 'feasible and beneficial to medical students’ [12].

Given the frequency with which junior doctors interpret echocardiography reports, responding doctors predictably highlighted the perceived importance of this skill. Within this context, it is concerning that only a small minority felt confident with this skill, reporting a sound understanding. Unconfident doctors with a poor perceived understanding of echocardiography interpretation and management of cardiac pathology may result in suboptimal patient care and patient safety concerns or increase demand on supervising senior staff. 

This being said, data may suggest satisfactory education on heart failure and the accompanying echocardiographic features, as doctors rated their baseline understanding and confidence of such features significantly higher than that of other pathologies/report features. This may also result from relatively high prevalence of heart failure and greater clinical exposure. 

Regarding application of knowledge, equal proportions of correct answers amongst FY and IMT doctors in the KFQs may suggest that the teaching was equally as effective in both groups. The success of the teaching programme is illustrated by significant increases in perceived understanding and confidence of junior doctors in almost all aspects of echocardiography assessed. Feedback suggests high relevance to FY and IMT roles, reinforcing the appropriateness of education on this topic. 

Qualitative data highlights the efficacy of the teaching and displays an appetite amongst junior doctors for a practical skills session in echocardiography. Many felt that this would aid their understanding of the different views and improve their ability to interpret images and reports.

As this study assesses the outcomes of single-site teaching session involving small numbers of participants, generalisability of the findings may be limited. Although the teaching was offered to all FY and IMT doctors in a hybrid format to increase accessibility, a proportion were unable to attend this training due to clinical commitments or leave.

## Conclusions

This work highlights an existing lack of confidence amongst junior doctors in echocardiography report interpretation and corresponding pathology interpretation. This low-cost teaching intervention has been shown to effectively address this.

Given the success of the teaching programme and the high learner satisfaction rate, this effective education model is appropriate for large-scale adoption for junior doctors. Collaboration with medical schools is recommended to aid delivery at an earlier stage, to better equip graduates. Formal integration of echocardiography into both the undergraduate and postgraduate curricula is advised, with attention being paid to report interpretation alongside the practical skills. This work identifies the need for the expansion of echocardiography education within healthcare training to better prepare students and doctors for practice. We hope this work paves the way for a wider-scale research into the provision and efficacy of echocardiography education.
